# A systematic review of therapeutic interventions to reduce acute and chronic post-surgical pain after amputation, thoracotomy or mastectomy*

**DOI:** 10.1002/ejp.567

**Published:** 2014-08-04

**Authors:** SR Humble, AJ Dalton, L Li

**Affiliations:** 1 Department of Anaesthetics and Pain Management, Charing Cross HospitalLondon, UK; 2 Peripheral Neuropathy Unit, Hammersmith Hospital Campus, Imperial College LondonDu Cane Road, London, UK; 3 Department of Anaesthetics and Pain Management, Ninewells Hospital and Medical SchoolDundee, UK

## Abstract

**Background:**

Perioperative neuropathic pain is under-recognized and often undertreated. Chronic pain may develop after any routine surgery, but it can have a far greater incidence after amputation, thoracotomy or mastectomy. The peak noxious barrage due to the neural trauma associated with these operations may be reduced in the perioperative period with the potential to reduce the risk of chronic pain.

**Databases and data treatment:**

A systematic review of the evidence for perioperative interventions reducing acute and chronic pain associated with amputation, mastectomy or thoracotomy.

**Results:**

Thirty-two randomized controlled trials met the inclusion criteria. Gabapentinoids reduced pain after mastectomy, but a single dose was ineffective for thoracotomy patients who had an epidural. Gabapentinoids were ineffective for vascular amputees with pre-existing chronic pain. Venlafaxine was associated with less chronic pain after mastectomy. Intravenous and topical lidocaine and perioperative EMLA (eutectic mixture of local anaesthetic) cream reduced the incidence of chronic pain after mastectomy, whereas local anaesthetic infiltration appeared ineffective. The majority of the trials investigating regional analgesia found it to be beneficial for chronic symptoms. Ketamine and intercostal cryoanalgesia offered no reduction in chronic pain. Total intravenous anaesthesia (TIVA) reduced the incidence of post-thoracotomy pain in one study, whereas high-dose remifentanil exacerbated chronic pain in another.

**Conclusions:**

Appropriate dose regimes of gabapentinoids, antidepressants, local anaesthetics and regional anaesthesia may potentially reduce the severity of both acute and chronic pain for patients. Ketamine was not effective at reducing chronic pain. Intercostal cryoanalgesia was not effective and has the potential to increase the risk of chronic pain. TIVA may be beneficial but the effects of opioids are unclear.

## 1. Background and objective

Chronic pain may occur in a minority of patients after minor operations, but the incidence is much greater after procedures that involve major nerve injury or transection. This systematic review examines the impact of specific interventions on the management of perioperative pain and associated chronic pain in high-risk operations such as amputation (50–80%), mastectomy (20–40%) and thoracotomy (30–50%; Kehlet et al., 2006; Macrae, 2008). Neuropathic pain is typically due to a damaged or dysfunctional nervous system (Woolf and Salter, 2000; Macintyre and Schug, 2007; Gray, 2008) and it may be characterized by sharp, shooting or burning sensations associated with sensory loss and paradoxical hypersensitivity (Macrae, 2008). The underlying pathophysiological processes are highly complex, but involve the interaction of the immune and nervous systems, which results in peripheral and central sensitization (Scholz and Woolf, 2007; Harvey and Dickenson, 2008). Neuropathic changes are traditionally associated with chronic pain, whereas acute pain has been considered as a protective nociceptive process. However, acute neuropathic pain is now also considered to be an eminently treatable but significantly under-recognized condition (Dworkin et al., 2007; Gray, 2008). The true incidence of acute neuropathic pain after surgery is uncertain (Gray, 2008), but the incidence of acute neuropathic phantom limb pain after amputation is greater than 50% (Jaeger and Maier, 1992; Nikolajsen et al., 1997). In addition, severe post-operative pain is associated with a far higher risk of developing chronic pain (Kalkmann et al., 2003; Bisgaard et al., 2005; Kehlet et al., 2006; Hanley et al., 2007). Therefore, prompt, aggressive management has the potential to make a substantial difference in the short term and, perhaps even more importantly in the long term as well before symptoms become entrenched. However, if perioperative pain management regimes only incorporate opioids, non-steroidal anti-inflammatory drugs (NSAIDs) and regional analgesia, then patients with acute neuropathic pain may be undertreated and therefore at significantly increased risk of developing chronic pain. Chronic pain is considered to be a complex biopsychosocial condition and a long-term multidisciplinary approach to management may be required for many cases. The therapeutic options available for established chronic pain have substantial limitations and problematic side effects; therefore, preventative techniques are of particular importance.

**Database**

- Medline (1946–January 2014), Embase (1974–January 2014) and the Cochrane Library.
- Thirty-two randomized controlled trials met the inclusion criteria.

**What does this review add?**

- Appropriate dose regimes of gabapentinoids, antidepressants, local anaesthetics and regional anaesthesia may potentially reduce pain.
- Ketamine and intercostal cryoanalgesia were not effective at reducing chronic pain.
- Total intravenous anaesthesia may be beneficial.

## 2. Databases and data treatment

### 2.1 Search strategy

A literature search was carried out using Medline (1946–January 2014), Embase (1974–January 2014) and the Cochrane Library. The research question was, ‘What is the evidence for the use of specific therapeutic interventions in the management of perioperative pain in procedures that have a high risk of chronic pain and whether these interventions reduce chronic post-surgical pain?’ We used a combination of keywords and subject headings. The subjects of interest included amputation, mastectomy, thoracotomy, perioperative, pain, neuropathic, chronic pain, pre-emptive, epidural, regional and the relevant drug names and classes. All subject headings were examined for relevant terms that are related to the types of operation performed and the types of intervention. Articles were limited to randomized controlled trials (RCTs) in adult human participants. Additional studies were identified by manually tracking references from published papers. On some occasions, where a manuscript was unavailable, the authors were contacted directly.

Studies were also excluded in which subjects were recruited after the perioperative period and those with follow-up periods of less than 2 months were excluded. Studies with high risk of bias as assessed by the Cochrane Risk of Bias Tool (Higgins and Green, 2011) were excluded. Additional studies were identified by manually tracking references from published papers. On some occasions, where a manuscript was unavailable, the authors were contacted directly.

### 2.2 Article selection

All of the trials were reviewed independently by each of the authors and consensus was reached as to the inclusion and interpretation of each individual trial. This review focused only on surgical procedures associated with a high incidence of chronic pain: amputation, mastectomy and thoracotomy. Studies investigating acute pain and chronic pain were analysed, whereas studies in which subjects were recruited after the perioperative week and those with follow-up periods of less than 2 months were excluded. Once all of the trials were collated and analysed, an assessment was made of the potential to perform a meta-analysis of the quantitative efficacy of specific interventions.

## 3. Results

The combined literature search identified 598 records and a further 6 additional records were identified through the manual tracking of references. After the duplicates were removed, 331 records were screened, and of these, 252 excluded as being clearly irrelevant. A total of 79 records were therefore assessed for eligibility and of these 47 were excluded (the most common reasons were as follows: not RCT, inappropriate study design, high risk of bias, inadequate size, not related to pain and/or chronic pain not assessed). Thirty-two trials met the eligibility criteria and were included in the qualitative analysis.

### 3.1 Study characteristics

Of the 32 trials, 5 (16%) studied amputation, 17 (53%) studied thoracotomy and 10 (31%) studied mastectomy. Details of all studies are summarized in Tables 1 and 2. The mean sample size was 89 (±12 SD) subjects (range 36–343). The mean duration of patient follow-up in the trials was 7 (±1 SD) months following the surgical procedure (range 2–15). Four of the trials had more than two study arms and (reflecting the complexity of clinical practice) 13 trials incorporated multiple interventions within the same study. The outcome measures varied between studies; the visual analogue scale (VAS; 0–100 mm) was used in 22 trials; a version of the numerical rating scale (NRS; 0–10) was used in 20 trials; and the verbal rating score (0–3 = nil/mild/moderate/severe) was used in four trials. Pain at rest and movement was assessed in nine trials and quantitative sensory testing was used in eight trials. A multidimensional score such as the McGill Pain Questionnaire was used in nine trials.

**Table 1:** Randomized controlled trials of therapeutic interventions to reduce acute and chronic postsurgical pain

	Δ Ac	Δ Chr	Study	Intervention	Op	Size	RoB	Outcomes
1	Y	Y	Fassoulaki et al. (2002)	Mexiletine 600 mg/day versus gabapentin 1200 mg/day versus placebo, for 10 days.	M	75	+	Gabapentin and mexiletine reduced VAS scores versus control over first postop week (by clinically and statistically meaningful amounts). Gabapentin and mexiletine reduced daily oral analgesic use versus control over first postop week (codeine: 65, 55 and 113 mg, respectively; paracetamol: 2.6, 2.3 and 4.9 g, respectively; *p* < 0.05). No difference in overall incidence of chronic pain at 3 months (gabapentin 54%, mexiletine 45%, control 58%). Burning pain less frequent at 3 months (gabapentin 5%, mexiletine 5%, control 29%, *p* = 0.033).
2	Y	Y	Fassoulaki et al. (2005)	Gabapentin 1600 mg/day, ropivacaine infiltration and topical local anaesthesia cream versus placebo	M	50	+	Gabapentin and local anaesthetic reduced VAS scores versus control over first 3 days postop (by clinically and statistically meaningful amounts). Gabapentin and local anaesthetic reduced daily oral analgesic use versus control (paracetamol/codeine: 1.0 tablets vs. 4.4 tablets; *p* = 0.02) Gabapentin and local anaesthetic reduced incidence of chronic pain at 3 months (45% vs. 82%; *p* = 0.03) although not statistically significant at 6 months (30% vs. 57%; *p* = 0.15).
3	−	N	Nikolajsen et al. (2006)	Gabapentin versus placebo for 30 days. 300 up to 2100 mg/day. All patients received perioperative epidural analgesia.	A	41	?	Acute pain was not assessed. Gabapentin had no effect on incidence of phantom limb pain in these vascular patients at 1 month (gabapentin 55%, control 53%; *p* = 0.88) or at 6 months (gabapentin 59%, control 50%; *p* = 0.59). Low intensity of phantom limb pain at 6 months in both groups VAS: (gabapentin: 10 out of 100, control: 7.5 out of 100; *p* = 0.56). Opioid consumption was not significantly different between the two groups. Large proportion of patients had chronic pain before amputation (median VNRS: gabapentin: 8 out of 10 and control: 8 out of 10). Longer duration of pre-amputation pain in the treatment group. Longer duration of epidural infusion in the control group (gabapentin: 73 h, control: 95 h; *p* = 0.01). Appears underpowered and baseline group differences.
4	N	N	Kinney et al. (2012)	Single dose of gabapentin 600 mg preoperatively versus placebo. All patients received perioperative epidural analgesia.	T	120	?	Single dose of gabapentin did not improve acute post-thoracotomy pain scores (VNRS out of 10) versus control in patients already receiving epidural analgesia (gabapentin: day 1 = 3.1, day 2 = 2.5; control: day 1 = 2.9, day 2 = 2.5; *p* > 0.05). There was no difference in analgesic use. Single dose of gabapentin did not change incidence of chronic post-thoracotomy pain versus control in patients already receiving epidural analgesia (gabapentin: 70%, control: 66%; *p* = 0.72). No other quantitative measure of chronic pain at 3 months (insufficiently detailed assessment at 3 months).
5	Y	Y	Amr and Yousef (2010)	Venlafaxine 37.5 mg/day versus gabapentin 300 mg/day versus placebo for 10 days	M	150	?	Gabapentin reduced VAS after movement by clinically significant amounts daily from day 2 to day 10 (*p* = 0.003). Venlafaxine reduced VAS after movement by clinically significant amounts daily from day 8 to day 10 (*p* = 0.002). Analgesic consumption was reduced by clinically and statistically significant amounts for gabapentin versus control every day from days 0–10. At 6 months, venlafaxine (but not gabapentin) reduced the incidence of burning pain by clinically and statistically significant amounts (venlafaxine = 2%, gabapentin = 12%, control = 22%; *p* = 0.0018). At 6 months, venlafaxine (but not gabapentin) reduced the incidence of stabbing/pricking pain by clinically and statistically significant amounts (venlafaxine = 14%, gabapentin = 32%, control = 40%; *p* < 0.05).
6	Y	N	Dualé et al. (2009)	Ketamine bolus (1 mg/kg) and infusion (1 mg/kg/h) for 24 h. No regional technique except single-dose intrapleural ropivacaine.	T	80	+	Ketamine reduced aggregated VAS (area under curve) at 24 h versus control (ketamine = 73, control = 88; *p* = 0.039). Morphine consumption in first 24 h was not significantly different (ketamine = 37 mg, control = 41 mg; *p* = 0.068). At 48 h, aggregated VAS (area under curve) was not significantly different (ketamine = 69, control = 73; *p* = 0.672). At 4 months, there was no difference in any measures of pain or analgesic consumption. The incidence of ongoing pain was the same for both ketamine and control groups (ketamine = 24%, control = 34%; *p* = 0.325).
7	N	N	Hayes et al. (2004)	Ketamine bolus then infusion 0.15 mg/kg/h for 72 h	A	45	+	On day 3, the incidence of phantom limb pain was no different (ketamine = 65%, control = 45%; *p* = 0.34), but stump pain was higher for the ketamine group (ketamine = 95%, control = 65%; *p* = 0.04). On day 6, the incidence of phantom limb pain was no different (ketamine = 55%, control = 50%; *p* = 1.0) and stump pain was no different (ketamine = 80%, control = 65%; *p* = 0.48). VNRS scores were not significantly different at day 3, day 6 or at 6 months for phantom or stump pain. At 6 months, the incidence of phantom limb pain was not significantly different between groups (ketamine = 47%, control = 71%; *p* = 0.28) and the incidence of stump pain was not significantly different (ketamine = 47%, control = 35%; *p* = 0.72). Appears underpowered.
8	N	N	Mendola et al. (2012)	Epidural levobupivacaine ± intravenous S+ ketamine infusion	T	66	?	S+ ketamine had no impact on VNRS during the acute post-operative period days 0–3. However, S+ ketamine reduced the number of patients requiring supplemental epidural boluses in the first 48 h (day 0: S+ ketamine = 25%, control = 50%; *p* = 0.04; day 1: S+ ketamine = 22%, control = 53%; *p* = 0.01; day 2: S+ ketamine = 22%, control = 23%; *p* = 0.9; day 3: S+ ketamine = 3%, control = 7%; *p* = 0.5). At 6 months, S+ ketamine infusion had no impact on the incidence of chronic pain (VNRS > 3: S+ ketamine = 3.4%, control = 0%; *p* = 0.46; NPSI > 1: S+ ketamine = 55%, control = 39%; *p* = 0.23).
9	N	N	Joseph et al. (2012)	Epidural ropivacaine ± intravenous ketamine infusion	T	60	+	Ketamine offered no clinically or statistically significant benefit on pain at rest or when coughing during the first 48 h. There was no clinically or statistically significant difference in rescue analgesic consumption or epidural ropivacaine consumption over the first 48 h. At 3 months, ketamine made no difference to VNRS at rest or with abduction (rest: ketamine VNRS = 1.1, control VNRS = 0.3; *p* = 0.385; abduction: ketamine VNRS = 1.3, control VNRS = 1.1, *p* = 0.589) or to analgesic consumption. Relatively large proportion lost to follow-up at 3 months.
10	Y	N	Wilson et al. (2008)	Epidural bupivacaine ± epidural ketamine infusion (vs. placebo)	A	53	+	Ketamine was associated with lower VAS than control during the first 48–72 h (ketamine = 0 mm, control = 17 mm; *p* = 0.03) and with less epidural top ups (ketamine = 1.8, control = 3.5; *p* = 0.04). Mean epidural infusion rate and duration was the same for both groups. At 12 months, ketamine had no significant impact on the incidence of stump or phantom pain (stump pain: ketamine = 21%, control = 33%; *p* = 0.68; phantom pain: ketamine = 50%, control = 40%; *p* = 0.87). Recruitment was stopped after only 35 patients due to unfavourable interim analysis.
11	−	N	Ryu et al. (2011)	Epidural levobupivacaine and fentanyl ± epidural ketamine	T	133	+	Acute pain was not assessed, although, at 2 weeks, epidural ketamine had no significant impact on median VAS (rest: ketamine = 25, control = 25; *p* = 0.73; movement: ketamine = 50, control = 50; *p* = 0.54). At 3 months, epidural ketamine had no significant impact on the incidence of chronic pain (rest: ketamine = 51%, control = 43%; *p* = 0.35; movement: ketamine = 68%, control = 74%; *p* = 0.46).
12	N	N	Ju et al., (2008)	Epidural analgesia versus intercostal nerve cryoanalgesia and morphine PCA	T	114	?	Cryoanalgesia had no statistically significant impact on acute VNRS during first 72 h compared with control (data not shown in publication). At 12 months, cryoanalgesia had no significant impact on the incidence of chronic pain (cryoanalgesia = 56%, control = 42%; *p* = 0.21) but it did increase the incidence of allodynia (cryoanalgesia = 15%, control = 0%; *p* = 0.03) and it increased interference with daily life (cryoanalgesia = 33%, control = 8%; *p* = 0.01).
13	N	N	Yang et al. (2004)	Epidural anaesthesia ± intercostal nerve cryoanalgesia	T	80	?	Cryoanalgesia had no impact on VAS at rest during the first post-operative week. It had no impact on VAS with movement during the first 6 post-operative days. Cryoanalgesia reduced VAS on the seventh day by a statistically significant amount, although this was not necessarily clinically significant (exact data not described). At 6 months, cryoanalgesia had no significant impact on incidence of chronic pain (cryoanalgesia = 40%, control = 30%; *p* = 0.482) or VNRS (cryoanalgesia: rest VNRS = 2, movement VNRS = 2; control: rest VNRS = 1.8, movement VNRS = 2; *p* > 0.05).
14	N	N	Mustola et al. (2011)	Epidural anaesthesia ± intercostal nerve cryoanalgesia	T	42	?	Cryoanalgesia had no benefit during the first post-operative week. Pain scores (VAS or VNRS) were recorded 22 times during the first post-operative week (too many secondary endpoints). There was a trend for worse pain scores with cryoanalgesia, which reached statistical significance on two occasions. At 6 months, cryoanalgesia had no significant impact on incidence of chronic pain during exercise (cryoanalgesia = 25%, control = 10%; statistical data not shown) and no statistically significant impact on VAS (cryoanalgesia: rest VAS = 6.3, movement VAS = 19.3; control: rest VAS = 2.2, movement VAS = 7.6; *p* > 0.05). Trial appears underpowered.
15	?	N	Sepsas et al. (2013)	Intercostal nerve cryoanalgesia versus control	T	50	?	Cryoanalgesia reduced rest pain during the first week post-operatively by statistically significant amounts. During the first week, VNRS scores were obtained 13 times. During the first 2 months, VNRS was obtained a total of 16 times and at every single time; cryoanalgesia had a statistically significant benefit over control. At 2 months, cryoanalgesia had a statistically significant impact on VNRS, but this did not appear clinically significant (cryoanalgesia VNRS = 0, control VNRS = 0.25; *p* = 0.01). There was also a statistically significant reduction in percentage of patients requiring analgesics at 2 months (cryoanalgesia = 0%, control = 72%; *p* < 0.0001). Outcome data appear highly inconsistent with other cryoanalgesia trials.
16	N	N	Gwak et al. (2004)	Fentanyl PCA ± intercostal nerve cryoanalgesia	T	50	?	Cryoanalgesia had no impact on acute pain VAS or analgesic consumption during the first post-operative week compared with control (data shown graphically). At 6 months, cryoanalgesia had no significant impact on the incidence of chronic pain compared with control (cryoanalgesia = 28%, control = 44%, *p* = NS).
17	N	N	Lu et al. (2013)	Intercostal nerve cryoanalgesia versus non-divided intercostal muscle flap (NIMF)	T	160	+	Cryoanalgesia had no statistically or clinically significant impact on daily VNRS or daily analgesic consumption during the first postoperative week compared with NIMF technique patients. At 12 months, cryoanalgesia was associated with higher VNRS (cryoanalgesia = 3.2, NIMF = 1.3; *p* < 0.01)
18	N	N	Cerfolio et al. (2003)	Subcutaneous infiltration of lidocaine versus saline (all patients had epidural)	T	119	+	Lidocaine infiltration had no significant impact on acute pain VNRS or VAS during the first 3 post-operative days. At 12 months, lidocaine infiltration had no significant impact on VNRS (lidocaine = 1, control = 0.85; *p* = 1.0).
19	?	N	Vigneau et al. (2011)	Ropivacaine wound infiltration versus isotonic saline	M	46	+	Ropivacaine infiltration reduced acute pain VAS significantly during the first 6 h post-operatively, but had no significant impact for the remainder of the first 3 post-operative days. At 2 months, ropivacaine infiltration had no significant impact on VAS (ropivacaine: rest VAS = 9.1, movement VAS = 20.2; control: rest VAS = 4.4, movement VAS = 21.4; *p* > 0.05) or quality of life score.
20	?	N	Albi-Feldzer et al. (2013)	Ropivacaine wound infiltration versus isotonic saline	M	236	+	Ropivacaine infiltration reduced acute pain VAS significantly at rest and with movement during the first 90 min post-operatively, but there was no significant difference in VAS after that. At 12 months, ropivacaine infiltration had no significant impact on Brief Pain Inventory (BPI) score (ropivacaine = 1.97, control = 1.7; *p* = 0.41). A similar proportion of patients reported BPI 3 or above in both groups (ropivacaine = 33.3%, control = 26.9%; *p* = 0.37).
21	N	N	Baudry et al. (2003)	Ropivacaine wound infiltration versus isotonic saline	M	81	?	Ropivacaine infiltration had no significant impact on acute pain VAS or analgesic consumption compared to the control group. At 15 months after surgery, ropivacaine infiltration had no significant impact on the incidence of chronic pain (ropivacaine = 55%, control = 33%; *p* = 0.19).
22	?	Y	Grigoras et al., 2012	Lidocaine 1.5 mg/kg intravenous bolus and infusion versus saline infusion	M	36	+	Intravenous lidocaine reduced rest pain VAS 4 h post-operatively (lidocaine = 11.8, control = 29.5; *p* = 0.012). However, lidocaine had no significant impact on VAS at rest or with movement at any other assessments during the first post-operative week. At 3 months, lidocaine significantly reduced the incidence of chronic pain (lidocaine = 11.8%, control = 47.4%; *p* = 0.031), the intensity of pain (lidocaine VAS = 2.6, control VAS = 14.6; *p* = 0.025) and the area of hyperalgesia (lidocaine = 0.2 cm, control = 3.2 cm; *p* = 0.002). Appears underpowered.
23	N	Y	Fassoulaki et al. (2000)	EMLA cream 5 g versus inactive placebo cream perioperatively for 5 days	M	45	+	Eutectic mixture of local anaesthetic (EMLA) cream had no significant impact on acute pain VAS during the first 6 days post-operatively (data shown graphically). At 3 months, the group that received EMLA had a statistically and clinically significantly reduced incidence of total chronic pain compared to control (EMLA = 43%, control = 91%; *p* = 0.002).
24	Y	Y	Obata et al. (1999)	Epidural mepivacaine infusion commenced 20 min prior to surgery versus at the end of the operation	T	70	+	Commencing the epidural prior to surgical incision caused a significant reduction in pain VAS at rest after 4 h post-operatively and on the second and third post-operative days (data expressed in graphical form only). Early epidural commencement offered no significant advantage for the rest of the first post-operative week. There was no difference in NSAID use between groups during the first week. At 6 months, commencing the epidural prior to surgical incision significantly reduced the incidence of chronic pain compared with commencing at the end of the operation (epidural before = 33%, epidural after = 67%; *p* = 0.01). No control arm without epidural.
25	Y	Y	Senturk et al. (2002)	PCA versus epidural pre- or post-surgical procedure	T	69	−	Commencing the epidural prior to surgical incision caused a statistically and clinically significant reduction in pain VNRS at rest, with movement and with coughing during the first 48 h post-operatively compared with the other two groups: late epidural commencement and intravenous patient-controlled analgesia (i.v. PCA). At 6 months, commencing the epidural prior to surgical incision was associated with the lowest incidence of chronic pain and this was statistically and clinically significant (epidural before = 45%, epidural after = 63%, i.v. PCA = 78%; *p* < 0.05 between early epidural and i.v. PCA). Patients not blinded to group allocation (although clinicians are reported to be blinded). Therefore plausible bias that raises some doubts about the results. Appears underpowered.
26	N	N	Ochroch et al. (2002)	Epidural analgesia commenced prior to incision or at the end of operation	T	157	?	Commencing the epidural prior to surgical incision had no significant impact on pain VAS during the first 5 post-operative days (data displayed graphically), but it did have an epidural sparing effect (epidural before = 499 mL, epidural after = 622 mL; *p* = 0.035). At 11 months, commencing the epidural prior to surgical incision appeared to offer no significant benefit over commencing the epidural at the end of the operation but before reversal of general anaesthesia (data only shown graphically). Significant gender imbalance between groups (epidural before 61% women, epidural after = 43% women; *p* = 0.047). Authors also demonstrate significantly worse BPI pain scores for women in general in this setting (data displayed graphically). Therefore plausible bias that raises some doubts about the results.
27	N	Y	Salengros et al. (2010)	Presurgical epidural and low-dose intraoperative remifentanil infusion versus post-surgical epidural and high-dose intraoperative remifentanil infusion	T	38	+	Commencing the epidural prior to surgical incision and using the lower concentration remifentanil infusion intraoperatively had no impact on acute pain VNRS total aggregate scores during hospital stay either at rest or with movement. Commencing the epidural prior to surgical incision and using the lower concentration remifentanil infusion intraoperatively caused a statistically and clinically significant reduction in the surface area of chest wall allodynia during the first 3 post-operative days and also one month later (data only shown graphically). At 9 months, commencing the epidural prior to surgical incision and using the lower concentration remifentanil infusion appeared to significantly reduce the incidence of chronic pain (epidural before = 16.7%, epidural after = 70%; *p* = 0.009) and also the mean chronic pain intensity (epidural before VNRS = 0.72, epidural after VNRS = 1.6; *p* = 0.013). Did not have a 3rd control arm without epidural. Appears underpowered.
28	–	N	Nikolajsen et al. (1997)	Epidural bupivacaine and morphine prior to amputation versus epidural saline with intramuscular or oral morphine	A	60	?	Acute pain not described in specific detail. At 12 months, epidural analgesia had no significant impact on the incidence of phantom limb pain (epidural = 75%, control = 69%; *p* = 1.0) or the intensity of phantom or stump pain VAS in these vasculopaths (epidural: phantom = 20 mm, stump = 0 mm, control: phantom = 9 mm, stump = 4 mm; *p* > 0.05). Appears underpowered and epidural group had higher median pain VAS and higher opioid consumption prior to operation, therefore plausible bias that raises some doubts about the results.
29	Y	Y	Kairaluoma et al. (2006)	Paravertebral block versus sham block	M	60	+	Paravertebral block caused a significant reduction in the number of patients reporting pain VAS greater than 3 out of 10 during the first 2 post-operative weeks (data only shown graphically). At 12 months, paravertebral block significantly reduced the prevalence of chronic pain (paravertebral block = 43%, control = 77%; *p* = 0.008) and the intensity of pain VNRS with movement (paravertebral block VNRS = 0, control VNRS = 2; *p* = 0.003).
30	N	Y	Ibarra et al. (2011)	General anaesthesia ± paravertebral block	M	40	−	Paravertebral block had no significant impact on acute pain VAS with movement compared to control (paravertebral block VAS: at 60 min = 3, at 24 h = 2.3; control VAS: at 60 min = 2.9, at 24 h = 2.8; *p* = NS). At 5 months, paravertebral block significantly reduced the prevalence of chronic pain (paravertebral block = 6.7%, control = 50%; *p* = 0.01). Underpowered. High proportion lost to follow-up. Unclear risk of bias for multiple domains.
31	Y	Y	Karanikolas et al. (2011)	RCT with five trial arms examining effect of different regimes incorporating perioperative epidural or fentanyl PCA versus ‘conventional analgesia’	A	65	+	Complex trial with 5 treatment arms including combinations epidural, i.v. PCA and control analgesia. The best post-operative VAS scores at 24 h were in the patients that received epidural pre-, during and post-amputation and those that had general anaesthesia with a PCA pre- and post-operatively (Epi/Epi/Epi = 0, PCA/Epi/Epi = 22.5, PCA/Epi/PCA = 17.5, PCA/GA/PCA = 0, control analgesia = 50; *p* = 0.001). At 6 months, the prevalence of chronic pain was lowest in the patients that received epidural pre-, during and post-amputation (Epi/Epi/Epi = 7.7%, PCA/Epi/Epi = 30.7%, PCA/Epi/PCA = 58.3%, PCA/GA/PCA = 23%, control analgesia = 75%; *p* = 0.004). Appears underpowered due to 5 treatment arms.
32	N	Y	Song et al. (2012)	Thoracic epidural with total intravenous anaesthesia versus thoracic epidural with inhalational anaesthesia	T	343	+	Total intravenous anaesthesia (TIVA) with propofol and remifentanil had no impact on acute pain VRNS after thoracotomy compared to inhalational anaesthesia with sevoflurane. At 6 months, the group that received TIVA had a statistically and clinically significant reduced incidence of chronic pain (TIVA = 33.5%, 50.6%, *p* = 0.002).

Randomized controlled trials (RCTs) are listed according to the type of intervention.

‘?’, unclear risk of bias for one or more key domains; ‘−’, high risk of bias for one or more key domains; ‘+’ low risk of bias for all key domains; Δ, potentially beneficial effect; A, amputation; Ac, acute; Chr, chronic; M, mastectomy; NSAIDs, non-steroidal anti-inflammatory drugs; Op, operation; PCA, patient-controlled analgesia; RoB, Cochrane Collaboration Risk of Bias; T, thoracotomy; VAS, visual analogue scale; VNRS, verbal numerical rating score.

**Table 2:** Cochrane Collaboration Risk of Bias assessment summary

	Study	Sequence generation	Allocation concealment	Blinding of participants and personnel	Blinding of outcome assessment	Incomplete outcome data	Selective reporting	Other bias	Overall risk of bias
1	Fassoulaki et al. (2002)	+	+	+	+	+	?	+	**+**
2	Fassoulaki et al. (2005)	+	+	+	+	+	?	+	**+**
3	Nikolajsen et al. (2006)	+	+	+	+	?	?	?	**?**
4	Kinney et al. (2012)	+	+	+	+	?	?	+	**?**
5	Amr and Yousef (2010)	+	+	+	+	?	?	+	**?**
6	Dualé et al. (2009)	+	+	+	+	?	+	+	**+**
7	Hayes et al. (2004)	+	+	+	+	+	?	+	**+**
8	Mendola et al. (2012)	+	+	+	+	+	?	+	**+**
9	Joseph et al. (2012)	+	+	+	+	?	+	+	**+**
10	Wilson et al. (2008)	+	+	+	+	?	+	+	**+**
11	Ryu et al. (2011)	+	+	+	+	?	+	+	**+**
12	Ju et al. (2008)	?	+	+	+	?	?	+	**?**
13	Yang et al. (2004)	+	+	+	+	?	?	+	**?**
14	Mustola et al. (2011)	?	+	+	+	?	?	+	**?**
15	Sepsas et al. (2013)	?	+	+	+	?	?	?	**?**
16	Gwak et al. (2004)	?	+	+	+	?	?	+	**?**
17	Lu et al. (2013)	+	+	+	+	+	?	+	**+**
18	Cerfolio et al. (2003)	?	+	+	+	+	?	+	**+**
19	Vigneau et al. (2011)	+	+	+	+	+	?	+	**+**
20	Albi-Feldzer et al. (2013)	+	+	+	+	+	?	+	**+**
21	Baudry et al. (2003)	+	+	+	+	?	?	+	**?**
22	Grigoras et al. (2012)	+	+	+	+	+	?	+	**+**
23	Fassoulaki et al. (2000)	?	+	+	+	+	?	+	**+**
24	Obata et al. (1999)	?	+	+	+	+	?	+	**+**
25	Senturk et al. (2002)	?	−	−	+	+	?	+	−
26	Ochroch et al. (2002)	?	+	+	+	+	?	+	**?**
27	Salengros et al. (2010)	?	+	+	+	+	?	+	**+**
28	Nikolajsen et al. (1997)	?	+	+	+	?	?	?	**?**
29	Kairaluoma et al. (2006)	+	+	+	+	+	?	+	**+**
30	Ibarra et al. (2011)	?	+	?	+	−	?	+	−
31	Karanikolas et al. (2011)	+	+	+	+	+	+	+	**+**
32	Song et al. (2012)	+	+	+	+	+	?	+	**+**

‘+’, low risk of bias (unlikely to seriously alter the results); ‘?’, unclear risk of bias (raises some doubt about the results); ‘−’, high risk of bias (seriously weakens confidence in the results). The far right column (in bold) gives overall risk of bias based on all the values within an entire row.

### 3.2 Interventions studied

Five of the trials studied the impact of the gabapentinoids and the dose regimen and duration of therapy was different in every trial (Fassoulaki et al., 2002, 2005; Nikolajsen et al., 2006; Amr and Yousef, 2010; Kinney et al., 2012). One trial incorporated the antidepressant venlafaxine (Amr and Yousef, 2010). Six trials studied the impact of ketamine; two consisted of intravenous ketamine without epidural analgesia (Hayes et al., 2004; Dualé et al., 2009), two consisted of intravenous ketamine in addition to epidural analgesia (Joseph et al., 2012; Mendola et al., 2012) and two examined the effect of epidural ketamine (Wilson et al., 2008; Ryu et al., 2011). Six trials studied the impact of cryoanalgesia in thoracotomy. Of these, two trials compared cryoanalgesia against control treatment with intravenous opioids (Gwak et al., 2004; Sepsas et al., 2013), one trial compared cryoanalgesia to epidural analgesia (Ju et al., 2008), two trials compared the effect of cryoanalgesia versus placebo in thoracotomy patients who all had epidural analgesia (Yang et al., 2004; Mustola et al., 2011) and one trial compared cryoanalgesia to non-divided intercostal muscle flap (Lu et al., 2013). Six trials examined the impact of local anaesthetic; of these, three incorporated infiltration with differing doses of ropivacaine in mastectomy (Albi-Feldzer et al., 2013; Baudry et al., 2003; Vigneau et al., 2011), one trial studied the impact of lidocaine infiltration in thoracotomy patients that already had a thoracic epidural *in situ* (Cerfolio et al., 2003), one trial examined the effect of intravenous lidocaine (Grigoras et al., 2012) and the remaining one assessed the impact of topical EMLA (eutectic mixture of local anaesthetic) cream in mastectomy patients (Fassoulaki et al., 2000). The impact of regional analgesia was studied in eight trials (two amputation, two mastectomy and four thoracotomy). Three trials examined whether commencing an epidural prior to thoracotomy was better than commencing it at the end of the procedure (Obata et al., 1999; Ochroch et al., 2002; Senturk et al., 2002). One trial compared pre-surgical epidural and low-dose intraoperative remifentanil infusion versus post-surgical epidural and high-dose intraoperative remifentanil infusion (Salengros et al., 2010). One trial compared epidural analgesia to epidural saline plus standard management of thoracotomy pain (Nikolajsen et al., 1997). Two trials examined the impact of paravertebral block for mastectomy patients (Kairaluoma et al., 2006; Ibarra et al., 2011). One trial had five study arms that studied epidural therapy, fentanyl patient-controlled analgesia (PCA) and standard therapy before, during and after amputation (Karanikolas et al., 2011). A single trial examined the impact of total intravenous anaesthesia (TIVA) versus inhalational anaesthesia in thoracotomy patients with thoracic epidurals *in situ* (Song et al., 2012).

### 3.3 Study findings

#### 3.3.1 Gabapentinoids and antidepressants

In patients after mastectomy, gabapentinoids reduced acute pain in three trials (Fassoulaki et al., 2002, 2005; Amr and Yousef, 2010). Gabapentinoids also reduced the overall incidence of chronic pain in one (Fassoulaki et al., 2002) and the incidence of chronic burning pain in another (Fassoulaki et al., 2005). Gabapentin did not reduce acute pain in two trials where epidural analgesia was used for amputation or thoracotomy, respectively (Nikolajsen et al., 2006; Kinney et al., 2012). In the former, therapeutic doses of gabapentin were used, but the patients had chronic pain preoperatively secondary to vascular disease. In the latter, only a single dose of gabapentin was given perioperatively. The antidepressant venlafaxine (37.5 mg) was associated with significantly lower post-mastectomy pain after 7 days, and after 6 months, the incidence of post-mastectomy burning and stabbing pain was significantly lower (Amr and Yousef, 2010).

#### 3.3.2 Ketamine

In one trial, intravenous ketamine infusion significantly reduced acute pain after thoracotomy but did not reduce chronic pain (Dualé et al., 2009). In three trials, intravenous ketamine had no impact on acute or chronic pain with or without the presence of epidural analgesia for amputation or thoracotomy (Hayes et al., 2004; Joseph et al., 2012; Mendola et al., 2012). Epidural ketamine was co-administered with bupivacaine in two trials. In one of these, ketamine reduced acute pain but not chronic pain (Wilson et al., 2008), and in the other trial, acute pain was not assessed, but the drug had no impact on chronic pain (Ryu et al., 2011).

#### 3.3.3 Cryoanalgesia

In one trial of thoracotomy patients, intercostal cryoanalgesia reduced acute pain versus control therapy (no epidural; Sepsas et al., 2013). In the same trial, there was a difference in pain scores at 2 months that was statistically significant, but not clinically significant (VAS: 0.0/10 vs. 0.25/10). However, in a further five thoracotomy trials, cryoanalgesia had no impact on acute pain (Gwak et al., 2004; Yang et al., 2004; Ju et al., 2008; Mustola et al., 2011; Lu et al., 2013). Of these five thoracotomy trials, intercostal cryoanalgesia had no impact on chronic pain in one trial (Gwak et al., 2004) and actually increased chronic pain in four trials (Yang et al., 2004; Ju et al., 2008; Mustola et al., 2011; Lu et al., 2013).

#### 3.3.4 Local anaesthetic

Six trials investigated the effect of local anaesthetics. Ropivacaine infiltration reduced acute pain for 1.5 and 6 h, respectively, in two trials (Vigneau et al., 2011; Albi-Feldzer et al., 2013), but had no impact on the development of chronic post-mastectomy pain. In a third ropivacaine trial, the drug had no impact on acute or chronic pain (Baudry et al., 2003). Lidocaine infiltration also had no impact on acute or chronic pain in thoracotomy patients (Cerfolio et al., 2003), whereas in one trial, intravenous lidocaine infusion (1.5 mg/kg pre- and intraoperative) had no impact on acute pain overall, but significantly reduced the incidence chronic pain and the area of hyperalgesia (Grigoras et al., 2012). In another trial, topical EMLA cream applied (for 5 days) had no impact on acute pain, but it reduced the incidence of chronic pain after mastectomy (Fassoulaki et al., 2000).

#### 3.3.5 Regional anaesthesia, remifentanil and propofol

Seven trials investigated the acute and chronic impact of regional anaesthesia including whether it could have a beneficial pre-emptive effect. One study found that although epidural analgesia was effective in the acute perioperative setting, it did not reduce chronic phantom limb pain in patients who had chronic pain preoperatively secondary to vascular disease (Nikolajsen et al., 1997). In contrast, two trials found that epidural analgesia reduced acute and chronic pain versus control therapy after thoracotomy and amputation, respectively (Senturk et al., 2002; Karanikolas et al., 2011). Four trials examined whether the timing of epidural analgesia could impact on acute and chronic pain (Obata et al., 1999; Ochroch et al., 2002; Senturk et al., 2002; Karanikolas et al., 2011). One of these trials found that it made no difference to acute or chronic pain whether the epidural was commenced immediately before or after the thoracotomy procedure (Ochroch et al., 2002). In contrast, the remaining three trials found that the optimization of analgesia preoperatively had a beneficial impact on both acute and chronic pain (Obata et al., 1999; Senturk et al., 2002; Karanikolas et al., 2011). Two trials of mastectomy patients investigated preoperative paravertebral block. The first trial found that paravertebral block significantly reduced acute pain and chronic pain 1 year later compared with sham block (Kairaluoma et al., 2006). The second trial found that paravertebral block had no significant impact on acute pain, but reduced the prevalence of chronic pain, compared with control (Ibarra et al., 2011). One trial compared preoperative epidural commencement with low-dose intravenous remifentanil infusion versus post-operative epidural commencement with a high-dose intravenous remifentanil infusion for thoracotomy patients (Salengros et al., 2010). They found no difference in acute pain, but high-dose remifentanil with a post-operative epidural was associated with a higher incidence of chronic pain. In addition, they found that the surface area of chest wall allodynia was significantly larger for the high-dose remifentanil group during the first post-operative month.

A single large trial of thoracotomy patients receiving thoracic epidurals compared TIVA with propofol and remifentanil against inhalational anaesthesia with sevoflurane (Song et al., 2012). There was no difference in acute pain between the two groups, but the incidence of chronic pain was significantly reduced at 3- and 6-month assessments in the TIVA group.

## 4. Conclusions

### 4.1 Principal findings

Gabapentinoids, antidepressants, local anaesthetics and regional anaesthesia have the potential to reduce the severity of both acute and chronic pain for patients undergoing amputation, thoracotomy or mastectomy. Ketamine and cryoanalgesia do not appear to be promising agents for reducing chronic pain. TIVA with propofol may have the potential to reduce the incidence of chronic pain, whereas the effect of opioids such as remifentanil is unclear.

### 4.2 Overview of findings and limitations

This systematic review was limited to amputation, mastectomy and thoracotomy because these operations are considered to be associated with a combination of nociceptive and neuropathic symptoms and carry a high risk of chronic pain. If it is accepted that the severity of acute pain is correlated with the risk of developing chronic pain, then it follows that the perioperative period is a logical target for interventions aimed at reducing or preventing the phenomenon. The positive and the negative results of this review are both important to guide future clinical practice. Specifically, it is useful to note the ineffective or harmful therapies so that they may be avoided and further trials of them may also be avoided.

The trials included in this systematic review were of varying quality, but addressed the majority of conventional methodological standards. However, the heterogeneity of the trial methodology and outcome measures ruled out the performance of a meaningful meta-analysis. Despite the lack of consistency and comparability between trials, several consistent themes emerge in the results. Acute pain in the perioperative period after amputation, mastectomy and thoracotomy appears to be a mix of nociceptive and neuropathic pain. The acute neuropathic component is potentially responsive to agents that work on neuropathic symptoms, but not nociceptive symptoms. The acute neuropathic component may be relatively resistant to conventional analgesics, but is potentially treatable, and if the correct intervention is utilized in the correct manner (dose/duration/timing), it has the potential to reduce the development of chronic pain. A number of trials included in this review studied a single, brief intervention, which was typically not enough to achieve long-term benefits, potentially due to the fact that the process of neuroplasticity that can continue for days or weeks post-operatively. The days following an operation appear to be as important as the day of the operation itself, i.e., the potential benefits of a pain-free initial 12 h could be made obsolete if the pain is severe thereafter. Furthermore, neuropathic symptoms may take several days to manifest post-operatively, thus emphasizing the importance of recognition and referral to a vigilant and cognizant acute pain service.

### 4.3 Comments on specific interventions

#### 4.3.1 Gabapentinoids, antidepressants

There were mixed results for the gabapentinoids; these agents reduced acute post-operative pain unless an effective epidural was present, in which case no significant difference was observed. Within the trials, gabapentinoids were often given once only or at sub-therapeutic doses. However, if an appropriate therapeutic dose was administered for a longer period of time, the efficacy appeared greater. This is in keeping with other studies of acute post-mastectomy pain reported in the literature (Dirks et al., 2003; Grover et al., 2009; Kim et al., 2011). Of note, vascular patients with chronic pain preoperatively continued to have chronic pain post-operatively despite gabapentin. Separately, the antidepressant venlafaxine was associated with enhanced analgesia 1 week after mastectomy and after 6 months despite being administered at the potentially sub-therapeutic dose of 37.5 mg.

#### 4.3.2 Local anaesthetics and regional anaesthesia

Interpretation of the trials examining the potential benefits of regional analgesia as a single group was challenging due to the inherent heterogeneity of trial methodology. Effective regional techniques in the hands of skilled clinicians can ablate the peak noxious barrage (Bonhomme et al., 2006; Wennervirta et al., 2011) of pain in the perioperative period and potentially minimize pathological neural plasticity implicated in the genesis of chronic pain (Woolf and Salter, 2000; Scholz and Woolf, 2007; Harvey and Dickenson, 2008; Ivani et al., 2011). Unsurprisingly, the majority of the trials included in this review found regional analgesia to be beneficial for reducing perioperative pain and subsequent chronic symptoms. The single trial that found epidural analgesia to be no better than standard analgesia comprised vascular patients with pre-existing chronic pain prior to amputation. It is perhaps unsurprising that a few days of epidural analgesia did not impact on the chronic symptomatology of these vasculopaths. Several trials examined the timing of regional intervention. In particular, the complex trial by Karanikolas et al. (2011) indicated the benefits of early epidural insertion and continued infusion for the entire perioperative period. However, the same trial also found that if patients were managed aggressively and effectively with systemic opioids, an acceptable outcome could also be achieved. Early commencement of regional analgesia may offer some benefits for post-operative pain and may have other advantages such as facilitation of balanced anaesthesia, minimization of sympathetic stress on the cardiovascular system and potential reduction of blood loss. They are also particularly useful in patients who are opioid tolerant. Optimal duration of regional analgesia is yet to be determined and potential benefits may be lost if epidural infusions are discontinued too early for logistical reasons.

Infiltration of local anaesthetic could provide a brief period of analgesia, but did not impact on the risk of developing chronic pain. In contrast, intraoperative intravenous lidocaine and perioperative EMLA cream reduced the incidence of chronic pain following mastectomy, without making a significant impact on acute pain.

#### 4.3.3 Remifentanil and propofol

Remifentanil infusions have become a popular component of balanced anaesthesia for a multitude of surgical procedures. However, the theoretical concept of opioid-induced hyperalgesia appeared to be confirmed in a relevant clinical setting in the trial by Salengros et al. (2010). Indeed, high-dose intraoperative remifentanil was also associated with an increased risk of chronic post-surgical pain. In contrast, TIVA with propofol and remifentanil was associated with a reduced incidence of chronic post-thoracotomy pain than inhalational anaesthesia with sevoflurane (Song et al., 2012). Given the phenomenon of opioid-induced hyperalgesia (Ballantyne and Mao, 2003; Angst and Clark, 2006; Raghavan et al., 2011), the potential benefit of TIVA is more likely to be propofol-mediated, potentially via glutamate, GABA_A_ and glycine receptors (Song et al., 2012).

#### 4.3.4 Ketamine and cryoanalgesia

While ketamine is an effective co-analgesic agent for acute pain and has the advantage that it may be used by patients in whom the oral route is unavailable, it did not reduce the risk of chronic post-surgical pain and its use in general is limited by dysphoric side effects. Ketamine also offered no additional benefit in the presence of effective epidural analgesia. Of even greater concern is intercostal nerve cryoanalgesia, which could arguably be renamed as ‘cryoneuralgia’ and perhaps be considered as an adjunctive therapy only in the setting of palliative care.

#### 4.3.5 Implications for clinical practice

Current mainstream perioperative management for amputation, thoracotomy and mastectomy is highly variable, but the choices that anaesthetists make may have long-term implications for patients under their care. Standard practices that rely primarily on opioids and NSAIDs for perioperative analgesia may be inadequate for patients at risk of neuropathic pain. Separately, techniques such as cryoanalgesia should be avoided. Gabapentinoids and antidepressants (which are not considered to be effective for nociceptive symptoms), as well as local anaesthetics and regional anaesthesia, may potentially reduce the severity of both acute and chronic pain for patients undergoing operations associated with a high risk of chronic post-surgical pain. However, some pharmacological agents may take a number of hours to be of benefit and therefore therapeutic dosages should be used for several weeks in many instances.

There have been promising results for the use of gabapentinoids in other conditions (Brogly et al., 2008; Buvanendran et al., 2010; Gray et al., 2011) but anti-neuropathic pain drugs have not been proven to be beneficial for surgical interventions that are predominantly associated with nociceptive pain such as minor gynaecological procedures. Cases of neuropathic pain may be discovered using simple screening tools routinely, e.g., DN4 or LANSS (Bennett, 2001; Bouhassira et al., 2005), and by identifying those who are experiencing a disproportionate level of pain. Appropriate interventions at this stage may provide superior analgesia and facilitate optimum recovery. Not all cases of chronic post-surgical neuropathic pain may be prevented, but the risk may be reduced. This may decrease the burden on society and the amount of financial resources spent on the management of chronic pain syndromes.

#### 4.3.6 Implications for future research

Future trials in this field should focus on conditions with a high risk of neuropathic pain, e.g., amputation, thoracotomy and mastectomy. They should use appropriately high dosages of medications, and therapy could commence prior to surgery and continue for several weeks. Patient follow-up should be for a minimum of 6 months and a much more comprehensive and multidimensional assessment made of the character and severity of pain at rest and movement with an emphasis on level of function and basic neurophysiological testing (Melzack, 1987; Wilder-Smith et al., 2003; Kehlet and Rathmell, 2010; Wildgaard et al., 2011).
